# Multifunctional graphene oxide/iron oxide nanoparticles for magnetic targeted drug delivery dual magnetic resonance/fluorescence imaging and cancer sensing

**DOI:** 10.1371/journal.pone.0217072

**Published:** 2019-06-06

**Authors:** Roberto Gonzalez-Rodriguez, Elizabeth Campbell, Anton Naumov

**Affiliations:** Department of Physics & Astronomy, Texas Christian University, Fort Worth, TX, United States of America; Consiglio Nazionale delle Ricerche, ITALY

## Abstract

Graphene Oxide (GO) has recently attracted substantial attention in biomedical field as an effective platform for biological sensing, tissue scaffolds and *in vitro* fluorescence imaging. However, the targeting modality and the capability of its *in vivo* detection have not been explored. To enhance the functionality of GO, we combine it with superparamagnetic iron oxide nanoparticles (Fe_3_O_4_ NPs) serving as a biocompatible magnetic drug delivery addends and magnetic resonance contrast agent for MRI. Synthesized GO-Fe_3_O_4_ conjugates have an average size of 260 nm and show low cytotoxicity comparable to that of GO. Fe_3_O_4_ nanoparticles provide superparamagnetic properties for magnetic targeted drug delivery allowing simple manipulation by the magnetic field and magnetic resonance imaging with high r_2_/r_1_ relaxivity ratios of ~10.7. GO-Fe_3_O_4_ retains pH-sensing capabilities of GO used in this work to detect cancer versus healthy environments *in vitro* and exhibits fluorescence in the visible for bioimaging. As a drug delivery platform GO-Fe_3_O_4_ shows successful fluorescence-tracked transport of hydrophobic doxorubicin non-covalently conjugated to GO with substantial loading and 2.5-fold improved efficacy. As a result, we propose GO-Fe_3_O_4_ nanoparticles as a novel multifunctional magnetic targeted platform for high efficacy drug delivery traced *in vitro* by GO fluorescence and *in vivo* via MRI capable of optical cancer detection.

## Introduction

Graphene is a gapless semiconductor that is now actively used in microelectronics and materials science.[1, 2] Due to complexity of scalable fabrication, its functional derivatives provide higher benefit for some of the applications. For instance graphene oxide (GO) due to its ease in production, water solubility and optical properties offers an advantageous alternative for applications in biomedicine and optoelectronics.[3–6] Graphitic surface in GO is derivatized with epoxy, hydroxyl and carboxyl groups, that allow it to form water suspensions stabilized by hydrogen bonds.[7–9] These functional groups perturb graphitic structure resulting into ~2eV band gaps enabling GO fluorescence in the visible.[10, 11] Additionally, GO has a high surface area available for functionalization and superior mechanical properties,[12, 13] which altogether makes it attractive for optoelectronics (LED devices and solar cells), tissue engineering and drug delivery.[14–18] GO is utilized as a basis for nanoscale sensors serving for the detection of small molecules such as NO_2_ in automovite emissions,[19] proteins,[20] influenza viral strains [21] and fluorescence-based pH-sensing that can be used to detect cancerous environments.[22] GO exhibits efficient internalization and stable fluorescence emission inside the cells, and has low cytotoxicity at the concentrations used in imaging.[22–24]. This makes GO a potential candidate for drug delivery and imaging *in vitro* or *ex vivo* concomitantly allowing for the cancer detection. However, the lack of targeting capabilities and the inability of *in vivo* tracking hampers the utilization of GO as an effective drug delivery system *in vivo*.

Here we develop and explore the properties of GO-Fe_3_O_4_ conjugates additionally allowing for magnetic targeted delivery and magnetic resonance imaging. This nanohybrid is intended to address the afore mentioned deficiencies of GO platform and altogether provide a novel multifunctional theranostic system. Superparamagnetic iron oxide nanoparticles (Fe_3_O_4_ NPs) have applications in biosensing, hypertermina, magnetic-assisted drug delivery and magnetic resonance imaging (MRI).[25–28] They exhibit very low cytotoxicity and are highly biocompatible in the iron-rich bloodstream.[29] MRI contrast agents based on Gd^3+^ or Mn^2+^ are well-studied and commercially available but show substantial toxic response. As an example, Gd^3+^ shows competitive inhibition of biological processes requiring Ca^2+^ which can result in heart failure. After utilizing Gd-based contrast agents, high deposition of Gd^3+^ have been found in skin, kidneys and brain.[30–32] Nephrogenic systemic fibrosis has been also linked to Gd^3+^ in patients with kidney diseases.[33, 34] Iron Oxide nanoparticles showing substantially higher biocompatibility and no toxic response in mice with low accumulation in liver and kidneys and clearance from plasma within 24 hrs, provide significant advantage over conventional contrast agents.[35, 36] The application of Fe_3_O_4_ as MRI contrast agent is mainly based on shortening T_2_ relaxation times of water molecules[37] which attributes it to the category of negative contrast agents. Several parameters can further affect relaxation times T_1_ or T_2_ in MRI contrast agents such as nanoparticle environments, surface coating, nanoparticle size and synergistic effects.[38–41] Thus, conjugation of Fe_3_O_4_ and GO holds a promise for the altered and potentially improved MRI capabilities of iron oxide.

GO-Fe_3_O_4_ conjugates synthesized to date are mainly utilized for the applications of removing pollutants such as heavy metals or organic molecules by magnetic separation or in lithium ion batteries.[42–45] Few studies suggest GO-Fe_3_O_4_ conjugates as a potential agent for magnetic resonance imaging, however only reporting T_2_ values rather than r_2_/r_1_ ratio, which is not enough for their assessment as MRI contrast agents.[46, 47] Several studies also report the use of GO-Fe_3_O_4_ conjugates for molecular imaging via attaching an external fluorophore and ligand-based targeted drug delivery.[48, 49] Here we propose a novel approach utilizing intrinsic GO emission for both imaging and optical cancer sensing as well as proposing iron oxide for both MRI imaging and magnetic targeting. Such synergistic multifunctional application of the components of GO-Fe_3_O_4_ conjugates provides an advantage of simplified structure (no extra targeting or fluorophores are needed to be attached) and potential for decreased toxic profile by avoiding additional toxicity derived from external molecular fluorophores.[50] Most importantly, this work combines MRI/fluorescence imaging, and targeted drug delivery in one molecular platform with a novel capability of optical cancer detection. Such multimodal agents can provide complementary data to diagnose diseases as well as allowing for better spatial resolution *in vivo* studies. In this work, we synthesize the afore mentioned GO-Fe_3_O_4_ conjugates and test their imaging, cancer detection and anticancer drug delivery capabilities *in vitro* in HeLa, MCF-7 and HEK-293 cells.

## Experimental reagents and instruments

### Materials

5 nm Fe_3_O_4_ were obtained from Cytodiagnostics, Graphene oxide (GO) from Goographene, Doxorubicin was obtained from Selleckchem, 3-Aminopropyltriethoxysilane (APTES) from Gelest Inc. The next chemicals were obtained from Sigma-Aldrich: N-(3-Dimethylaminopropyl)-N′-ethylcarbodiimide hydrochloride (EDC), N-Hydroxysuccinimide (NHS), Hydrochloric acid (HCl), Sodium hydroxide (NaOH), Ammonium acetate, Ferrozine, Iron chloride (FeCl_2_), Hydroxylammonium chloride (HONH_2_HCl), Toluene.

### Preparation of graphene oxide–iron oxide nanocomposite

5nm Fe_3_O_4_ staring material was first activated for 4 hrs with APTES dissolved at 1% w/v in toluene. Activated Fe_3_O_4_ NPs were washed with toluene to remove free APTES, sedimented via centrifugation and finally dispersed in water. APTES-functionalized iron oxide was further coupled with graphene oxide (GO). Graphene Oxide was dispersed in DI water at 450 μg/mL and ultrasonically treated for 30 to 60 min at 3W to decrease the size of GO flakes down to 250 nm for effective cell internalization. Treated GO and Fe_3_O_4_ NPs were coupled in a conjugation reaction using 1mmol (EDC) and 1mmol of (NHS). After 6 hrs, samples were centrifugally washed with DI water three times to purify the product sedimented during the centrifugation. Acidity (pH 6.4) of GO suspension allows to run the coupling reaction with EDC in water without the presence of the buffer. It is reported that the reaction is less effective at higher pH, however in the pH range of 4.5 to 7.2 reaction was shown to take place.[51]. There are also some reports of this type of conjugation without buffer at pH 7. [52]

### Characterization

Synthesized GO-Fe_3_O_4_ conjugates were further characterized with Transmission Electron Microscopy (TEM JEOL JEM-2100) at 200 kV to assess the morphology, crystallinity and lattice spacing. Capacity of GO-Fe_3_O_4_ as an MRI contrast agent was assessed via measuring relaxation times T_1_ and T_2_ with Bruker (Minispec mq60) Relaxometer at 1.41 T at 37°C. This material took 18 seconds to bring all the material to the cube side by using a magnet. Fluorescence spectra of the nanoconjugates were measured with Horiba Scientific, SPEX NanoLog Spectrafluorometer with 400 nm excitation and the emission in the range of 420 to 762 nm. This emission was assessed at different pH conditions that were achieved by adding microliter aliquots of NaOH or HCl to yield pH in the range of 6 to 8.4.

Ferrozine assay was used to determine the iron concentration in this composite. In this assay 500 μL of aqueous GO-Fe_3_O_4_ suspension was mixed with 500 μL of 12M HCl to dissolve Fe_3_O_4_ NPs, 500 μL of 12M of NaOH to neutralize the solution and then with 120 uL of 2.8 M HONH_2_HCl in 4 M HCl, 50 μL of 10M Ammonium Acetate and 300 μL of 10 mM Ferrozine in 0.1M Ammonium Acetate to allow for the assessment of the iron content. Absorbance was measured at 562 nm using Agilent Technologies Cary 60 UV-Vis and compared with previously measured calibration curve constructed with FeCl_2_ as a standard.

### Doxorubicin complexation

Doxorubicin was complexed with GO-Fe_3_O_4_ noncovalently at a concentration of 25.5 μg/mL by overnight coincubation with prior vortexing. Bound DOX-GO-Fe_3_O_4_ nanocomposites were separated from uncomplexed drug with a magnetic field. Absorption spectra of the uncomplexed drug were used to find the concentration of that (16.5 μg/ml) and assess the percentage of the drug that got complexed representing loading efficiency. Starting with DOX concentration of 42 μg/ml therefore allows to load 25.5 μg/ml or 60.7% of the free DOX on GO-Fe_3_O_4_. Provided the stock GO-Fe_3_O_4_ concentration of 127 μg/ml used for complexion and assessed via GO characteristic absorption, the loading of DOX onto GO-Fe_3_O_4_ was calculated to reach 20 wt%.

### Cellular uptake and imaging

*In vitro* imaging was performed in three different cell types: HEK-293 (Human embryonic kidney fibroblast), HeLa (Human cervical carcinoma) and MCF-7 (Human breast cancer). GO-Fe_3_O_4_ or DOX-GO-Fe_3_O_4_ formulations were introduced to cells at concentration of 15 μg/mL and analyzed at several time points ranging from 30 min to 27h. Internalization study was performed in HeLa cells washed PBS and fixed with 4% paraformaldehyde at 30 min, 1, 3, 12, 24- and 27-hours. For the pH-based detection of cancer versus healthy cells, HEK-293, MCF-7 and HeLa cells were treated with 15 μg/mL of GO-Fe_3_O_4_, the concentration was measured using freeze-drying and verified via absorption measurements. Although the conjugates themselves were not sterilized, however all other materials (solvents and glassware) used were sterile. The location of each formulation was assessed using the intrinsic GO-Fe_3_O_4_ fluorescence emission in the visible. Images were taken with Olympus IX73 microscope coupled to photometrics camera PRIM 958. For internalization studies 480nm excitation and 535nm emission filters were used to selectively image GO-Fe_3_O_4_ conjugates whereas for cancer detection study we utilized 480nm excitation for 535 nm green emission and 550nm excitation for 635 nm red emission. Location of GO inside the cells is not considered during the calculation of intracellular green/red ratios, rather, the signal from all inside the cell is accumulated. Over 100 cells were analyzed to yield the aforementioned green/red ratios representing pH-sensing by the GO. Extracellular emission from GO was collected only from the samples that were not fixed and the medium was not replaced leaving all extracellular GO intact.

### Cytotoxicity assays

MTT cytotoxicity assays were performed with 3 formulations: GO-Fe_3_O_4_, DOX-GO-Fe_3_O_4_ and free DOX at the same concentrations of DOX derived from loading on DOX-GO-Fe_3_O_4_ and up to 15 μg/mL–imaging concentration of GO-Fe_3_O_4_. This assay was used to detect metabolic activity in cells based in a colorimetric probe. MTT assay test is based on the ability of living cells to reduce tetrazole (yellow) to formazan (purple) with the mitochondrial reductase; cell survival rates care calculated based on the absorbance with the formazan formed. For each concentration tested in MTT assay we used four replicas to calculate the error bars. Two technical replicas were performed in each MTT assay. Two cancer cell lines (HeLa–Human cervical carcinoma, and MCF-7 –Human breast cancer) were used in this work, as well as one non-cancer cell line (HEK-293, Human embryonic kidney fibroblast). Cells were obtained from ATCC and maintained in a Thermo-Scientific Midi 40 CO2 Incubator at 37.1°C with 5% carbon dioxide, 95% air.

## Results and discussion

### Structural characterization

**Fig 1:**
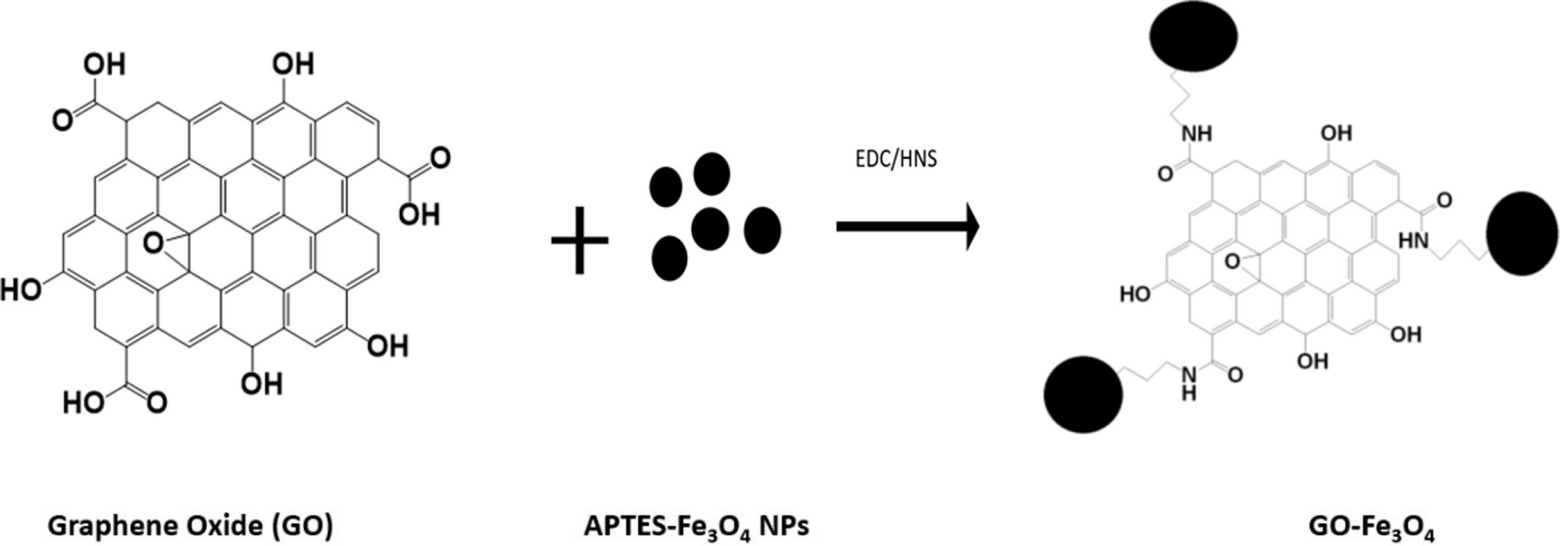
Representative schematic of GO-Fe_3_O_4_ conjugates formation.

**Fig 2:**
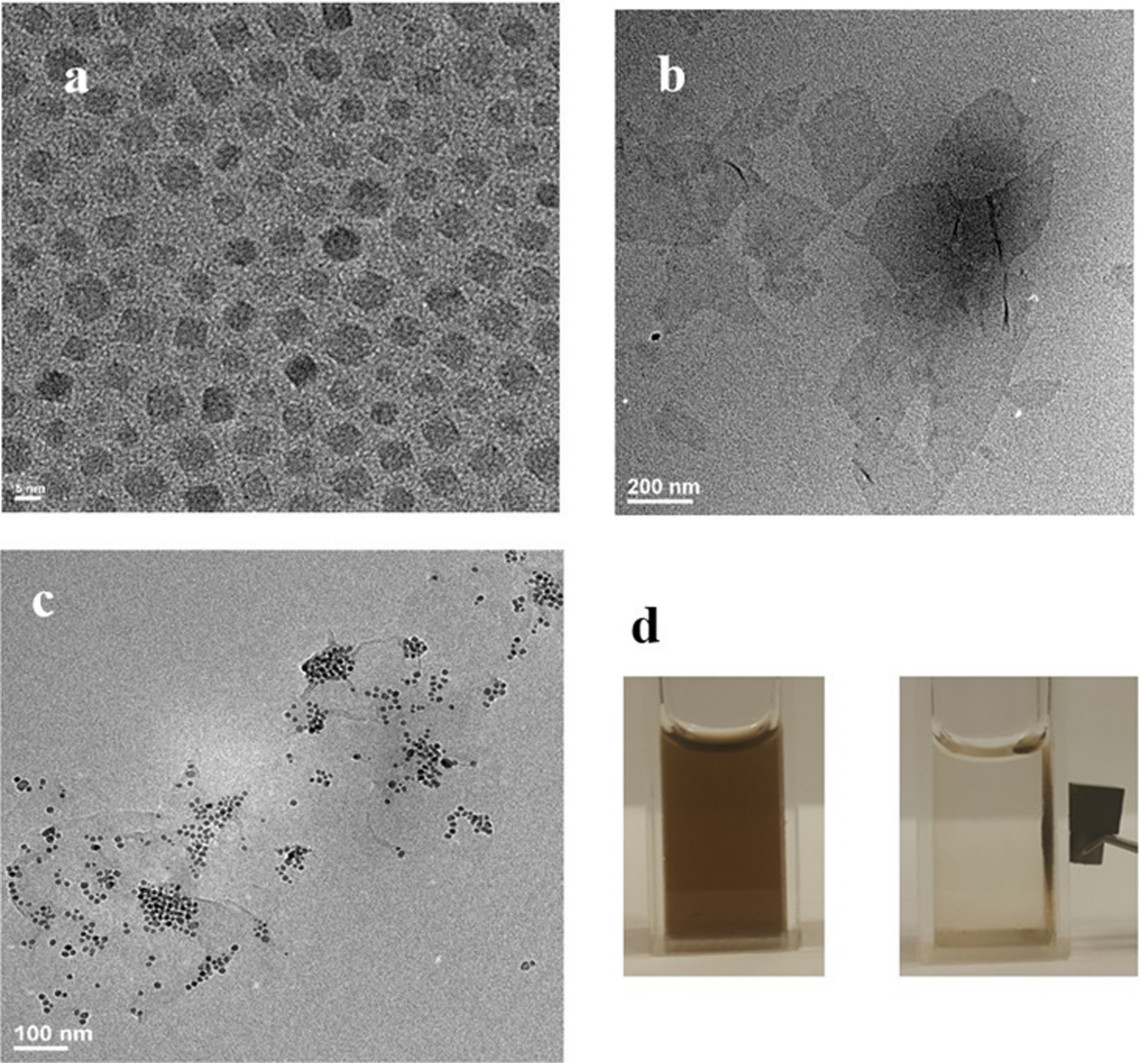
TEM of a) superparamagnetic Fe_3_O_4_ NPs, b) graphene oxide, c) GO-Fe_3_O_4_ conjugates and d) image of GO-Fe_3_O_4_ conjugates manipulated in solution by the of a magnetic field.

The formation of GO-Fe_3_O_4_ hybrids is achieved by a straightforward coupling reaction between superparamagnetic APTES-Fe_3_O_4_ NPs and GO in the presence of coupling reagents EDC and NHS (Fig 1) Prior to coupling, GO flakes are ultrasonically processed to reduce flake size for effective cellular internalization.[22] After 30 minutes of ultrasonic treatment GO flakes are reduced from micron-sized structures to an average size of 569 ± 310 nm (S1 Fig) and after 1 hour of treatment—to 257 ± 120 nm (Fig 2B). Although to fully predict the capability of intracellular transport the charge and hydrophobicity of GO need to be taken in account, the smaller ~250 nm nanoparticle sizes are expected to be more suitable for cellular internalization.[53–55] Fe_3_O_4_ nanoparticles coated with oleic acid used for coupling with GO show a uniform distribution and good dispersion with an average size of 5.8 nm (Figs 2A and S5A). As determined by HRTEM these nanoparticles have a lattice spacing of d = 0.29 nm (S5C Fig) corresponding to the spacing between (220) planes in magnetite. Coupling of Fe_3_O_4_ NPs with GO is achieved by functionalizing those with APTES that has an amino group reacting with carboxylic groups of GO in the presence of EDC/HNS. APTES replaces the oleic acid coating of Fe3O4 by ligand exchange. APTES is more stable than oleic acid due to a covalent bond between APTES and Fe3O4, whereas oleic acid is bonded by a noncovalent interaction The TEM of the final product, GO-Fe_3_O_4_ conjugates shows a randomly distributed Fe_3_O_4_ NPs across GO flakes (Fig 2C) while the Ferrozine assay complementary confirms the presence of iron. This verifies the success of the coupling reaction. Although we do not expect coupling to significantly affect GO flake sizes, dynamic light scattering (DLS) of GO-Fe_3_O_4_ conjugates (S7 Fig), yields mean size of 76 nm, as due to planar geometry of GO flakes DLS may not provide an accurate measurement of the flake dimensions. Thus, we verify the conjugate sizes with TEM statistical measurements of over 500 flakes yielding a mean size of 265 nm (S1C Fig). Zeta-potential of -3.18 ± 1.07 mV (S8 Fig), confirm that GO-Fe_3_O_4_ a negative charge of the conjugates as suspended particles. No precipitation of GO-Fe_3_O_4_ is observed in over a day in several media such as water, PBS, cell medium and serum (S6 Fig) indicating suspension stability of the conjugates. Following one-month shelf life the suspension of GO-Fe_3_O_4_ conjugates in water appeared stable with no observable precipitation. In 6 months, minimal amount of precipitate formed and was redispersed by 2 s of ultrasonic tip processing.

In this work we evaluate the capacity of synthesized GO-Fe_3_O_4_ conjugates for biomedical applications. We explore their ability to be manipulated by magnetic field for magnetic targeted therapy, their role as MRI contrast agents, fluorescence imaging capacity, the capability of cancer detection via optical pH-sensing and anticancer drug delivery.

### Magnetic targeting and MRI contrast agent capabilities

As synthesized hybrids show pronounced magnetic behavior and can be manipulated in suspension via a regular magnet (Fig 2D). This indicates a potential for magnetic targeting to the organs that require increased uptake of the delivered therapeutic. Although magnetic delivery to animal models was not explored, this is likely to take place due to high responsiveness of the nanoconjugates to the magnetic field (shown in Fig 2D), and may be object of future investigations. For the specific application of magnetic resonance imaging, the quality of an MRI contrast agent is more precisely evaluated by the relaxivity parameters r_1_ or r_2_, which describe the ability of a contrast agent to shorten the T_1_ or T_2_ relaxation times of water, rather than by T_1_ and T_2_ themselves. Thus, for GO-Fe_3_O_4_ conjugates we evaluate longitudinal r_1_ and transverse r_2_ relaxivity. These values are calculated through the dependence between the inverse proton relaxation times and the iron concentration:

$$\frac{1}{T_{i,obs}}=\frac{1}{T_{i,0}}+r_{i}\left[Fe\right]$$ (1)

**Fig 3:**
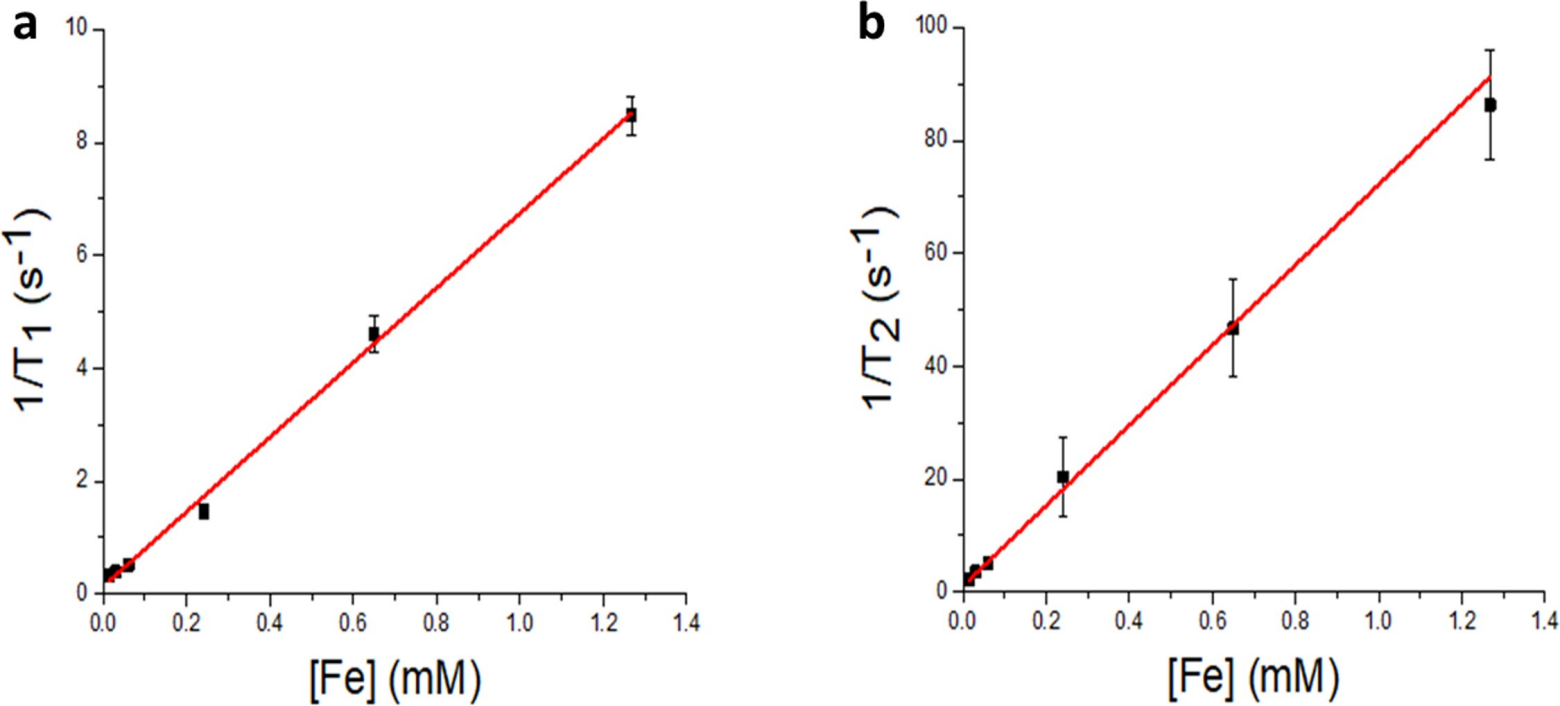
a) 1/T_1_ vs iron concentration [Fe] of GO-Fe_3_O_4_ conjugates and b) 1/T_2_ vs [Fe] of GO-Fe_3_O_4_ conjugates, the bars represent the standard deviation.

In this equation, 1/T_i,obs_ (i = 1,2) is the inverse relaxation time measured experimentally in the presence of iron oxide nanoparticles and 1/T_i,0_ is the inverse relaxation time of pure water in the absence of the contrast agent (GO-Fe_3_O_4_). r_i_ (i = 1,2) here is the longitudinal or transverse relaxivity and [Fe] is the iron concentration in GO-Fe_3_O_4_ nanoparticles.[56] The plot of relaxation rates 1/T_1_ and 1/T_2_ versus Fe concentration allows obtaining r_1_ = 6.6 mM^-1^s^-1^ and r_2_ = 71.1 mM^-1^s^-1^, with a ratio r_2_/r_1_ = 10.7 for GO-Fe_3_O_4_ (Fig 3) versus r_1_ = 15.7 mM^-1^s^-1^ and r_2_ = 36.2 mM^-1^s^-1^ and a ratio of r_2_/r_1_ = 2.3 for free Fe_3_O_4_ NPs control (S3 Fig). This is indicative of significant improvement for GO-Fe_3_O_4_ conjugates over uncomplexed Fe_3_O_4_ with the relaxivity ratio of r_2_/r_1_>2, placing them in the category of negative contrast agents. As compared to individual Fe_3_O_4_-based nanoparticles with reported highest r_2_/r_1_ ratios of 6.58[57] and 5.3[58] Fe_3_O_4_ conjugated to GO shows in this work a substantially higher potential for MRI imaging. A decreased r_1_ value for GO-Fe_3_O_4_ conjugates can be dictated by decreased access of water molecules to Fe_3_O_4_ partially obstructed by the GO, whereas the higher r_2_ value can be explained either by the similar interactions with GO or by formation of Fe_3_O_4_ NPs clusters on GO surface observed previously for free-standing Fe_3_O_4_ nanoparticles.[59–63] These NPs show minimal coercivity (Hc~ 50 Oe) at T = 300 K being far above T_B_, which means that no magnetic remanence is present and thus the magnetization of the samples vanishes if the applied magnetic field is switched off.[64, 65]

### Fluorescence imaging and pH-sensing

**Fig 4:**
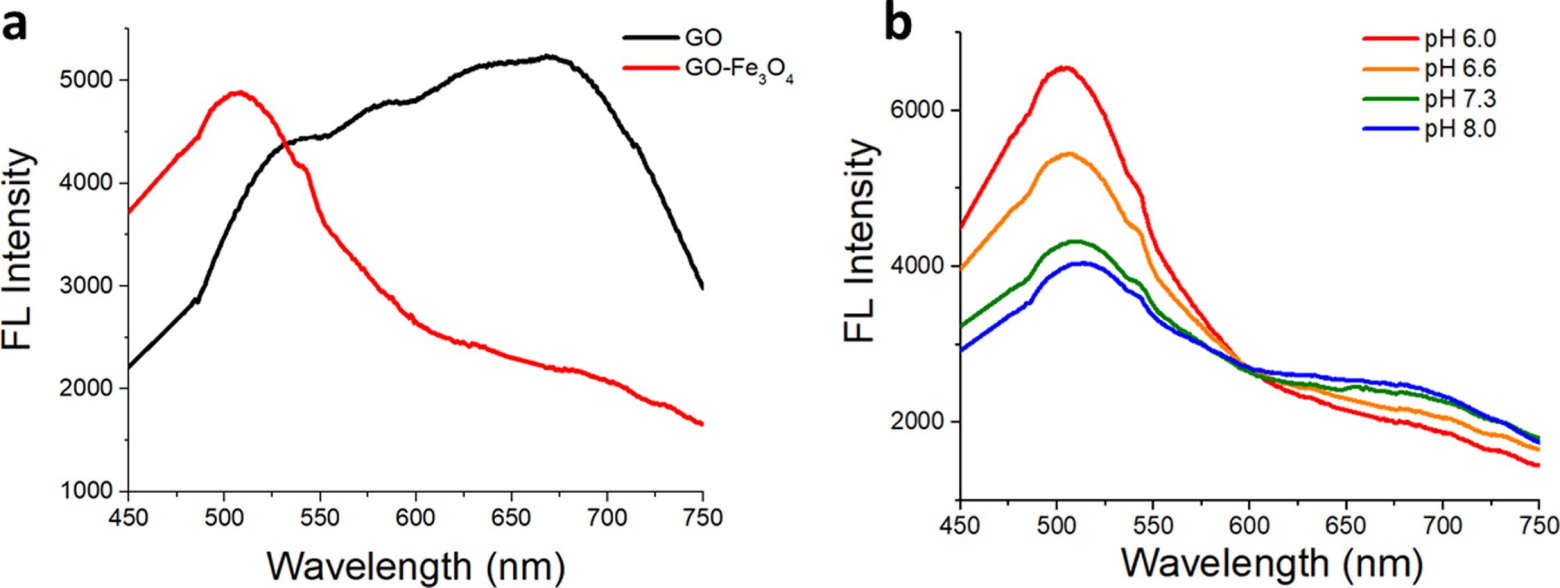
a) Fluorescence spectra of GO and GO-Fe_3_O_4_ conjugates b) pH fluorescence dependence of GO-Fe_3_O_4_ conjugates.

GO fluorescence emission detected in red/near-IR for the starting material[66] (Fig 4A) has experienced a substantial spectral change upon functionalization with Fe_3_O_4_ showing a narrower feature centered at 500nm with a broad shoulder in the red/near-IR. Notably the emission intensity was not affected by the functionalization still suggesting GO-Fe_3_O_4_ conjugates as effective candidates for *in vitro* fluorescence imaging. The emission is stable over several weeks and does not exhibit photobleaching or aggregation-related broadening.

As well as GO,[67] GO-Fe_3_O_4_ conjugates exhibit pH response in their emission. However, unlike GO, the increase in pH from 6 to 8 here results into quenching of the 500nm feature with subsequent slight enhancement in the red/near-IR shoulder and an isosbestic point at 600nm. This is indicative of the spectraphotometric titration behavior that in GO[67] was attributed to protonation/deprotonation of functional groups affecting electronic environments surrounding those. The ratios of green/red (500nm/650nm) GO-Fe_3_O_4_ emission intensities are calculated to be unique for each pH (S1 Table) providing the capability of pH-sensing on the nanoscale via an optical non-destructive method. This is highly applicable to cancer detection as cancerous environments are expected to have lower pH due to overexcretion of lactic acid by several cancer cell types.[68]

### In vitro imaging and cancer detection

**Fig 5:**
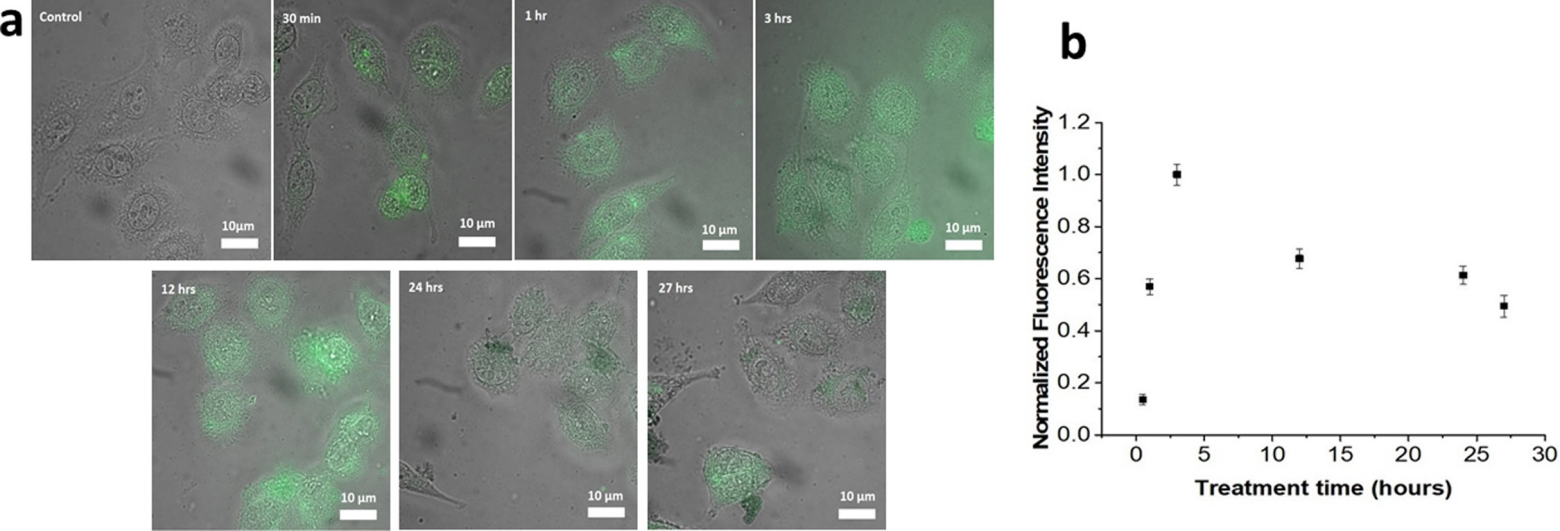
a) Images of GO-Fe_3_O_4_ fluorescence in HeLa cells at different transfection times and b) GO internalization over time assessed by average normalized intensity per unit emissive area of GO-Fe_3_O_4_ fluorescence in HeLa cells.

GO-Fe_3_O_4_ introduced to HeLa cells exhibits observable green (532 nm) emission, at 30 min, 1, 3, 12, 24 and 27 hours post transfection (Fig 5A). At each time point the emission intensity is significantly above the autofluorescence background in control samples and can be detected intracellularly. Extracellular GO-Fe_3_O_4_ is removed by repeated replacement when the cells are fixed with paraformaldehyde. In order to assess the optimal internalization time, we analyze over 100 cells at each time point for average emission intensity per unit emissive area. Intracellular emission is maximized at 3h post transfection (Fig 5B) indicating the optimal internalization timeline with the following decline. As GO shows no appreciable degradation or emission quenching over these time periods in cellular media (S4 Fig), we attribute intracellular emission decrease (Fig 5B) to slow excretion of GO-Fe_3_O_4_ conjugates over time down to 47% of the maximum in 27h. thus a high number of cells (100) per time point was used for internalization analysis.

**Fig 6:**
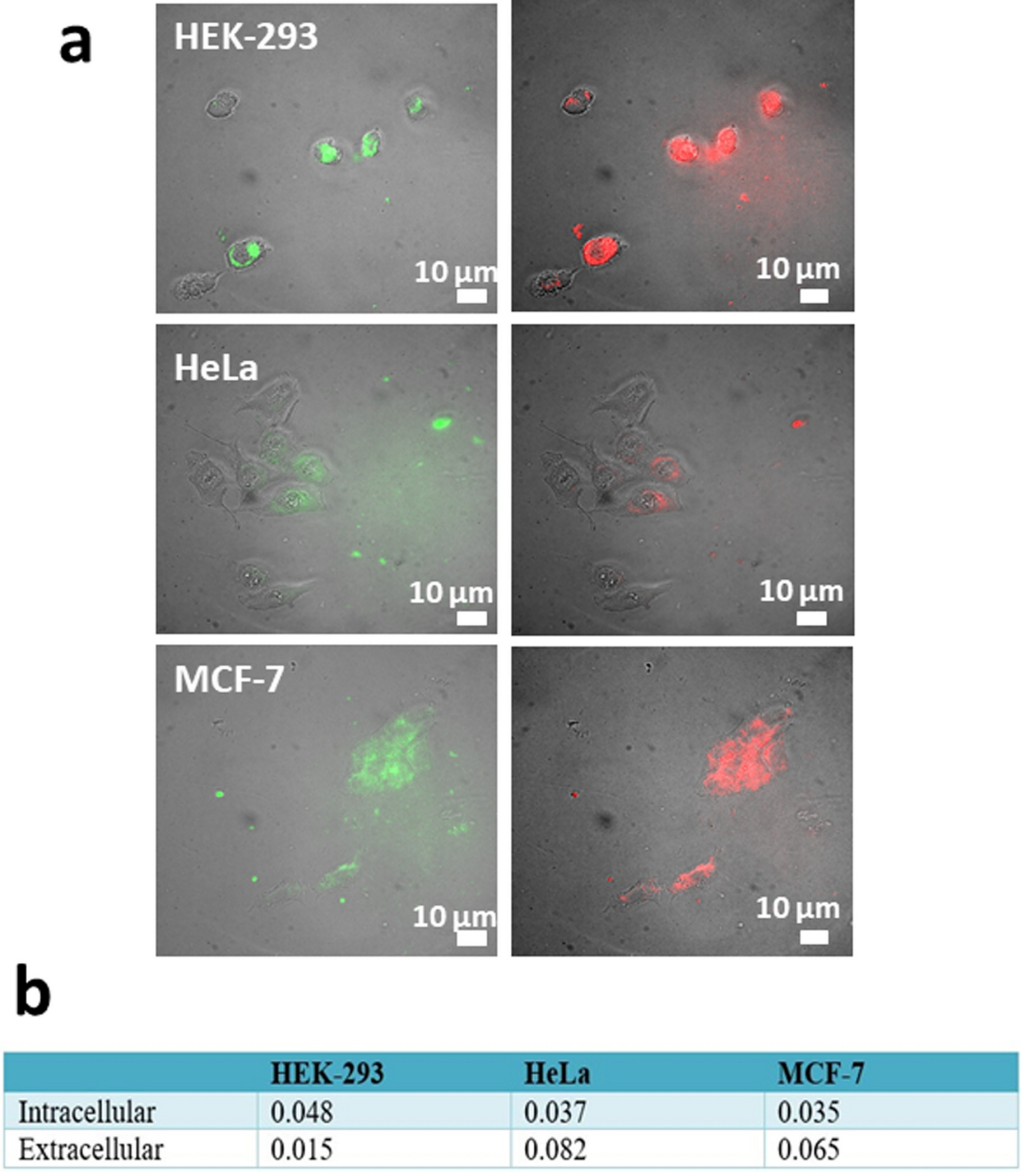
a) Images of GO-Fe_3_O_4_ emission in green (550 nm) and red (635 nm) in healthy HEK-293 versus cancer HeLa and MCF-7 cells b) Comparison of intracellular vs extracellular green/red ratios in healthy vs cancer cells.

We further utilize pH-dependence of GO-Fe_3_O_4_ emission to assess its cancer detection capability for cancer (HeLa and MCF-7) versus healthy (HEK-293) cellular environments *in vitro*. In order to account for potential variation of pH in different cancer cell environments we use two types of cancer cells and integrate the emission intensities in over a 100 fluorescence images to calculate average emission intensity per unit area at two different wavelengths in green and red. We anticipate that the number of cancer cells producing lactic acid would affect the capability of pH sensing by GO due to accessibility of all GO flakes to the acidic environments. Thus, we analyze over hundreds of cells to average out the response from those that may not be in equivalent environments within the imaging areas. Additionally, for our imaging experiments we estimate the cell density of 625 cells/mm^2^ that is within the standard cell density range used for *in vitro* work,[69, 70] indicating that pH sensing can be conducted using GO-Fe_3_O_4_ conjugates in regular *in vitro* experiments. Unlike in the internalization study, here we refrain from replacing the medium and focus mostly on extracellular emission of GO-Fe_3_O_4_ due to more complex pH environments inside the cells often subject to intracellular pH buffering. For pH sensing cells are not fixed thus allowing for GO to be present extracellularly. GO-Fe_3_O_4_ emission in red (635 nm) and green (535 nm) recorded in every cancer and healthy cell line with the spectrally-filtered microscopy imaging system providing characteristic green/red intensity ratios for pH assessment. These green/red emission intensity ratios show observable differences for cancer versus healthy cells (Fig 6A) which is confirmed by statistical measurements over the ensemble of cells (Fig 6B). Here higher ratios are observed for more acidic cancer cell environments as expected from the spectral dependence (Fig 4B). The very magnitudes of the intracellular emission-derived ratios can differ from the ones calculated from spectral pH behavior, since emission in microscopy images is recorded within the range of spectral filters. However, the general trend of higher green/red ratios for acidic environments of cancer cells prevails with 4–5 fold difference between cancer and healthy cells. Such significant detection ratio suggests a promising potential of GO-Fe_3_O_4_ as optical pH-sensors of cancerous environments.

### Drug delivery

The primary purpose of dual fluorescence/MRI imaging and pH-sensing capabilities of GO-Fe_3_O_4_ conjugates is to track anticancer drug delivery and image therapeutics in biological cells and tissues while allowing for concomitant cancer detection. We assess the drug transport properties of GO-Fe_3_O_4_ via the delivery of Doxorubicin non-covalently attached to GO surface. Doxorubicin (DOX) is an established chemotherapeutic that due to poor water solubility[71] has a need for nanocarrier delivery.[72, 73] Its hydrophobic structure advantageously allows DOX to complex non-covalently with several drug delivery vehicles including carbon nanotubes and polymeric micelles.[74–76] Non-covalent functionalization may facilitate improved drug release and is known to preserve optical/electronic properties of the nanocarrier essential for imaging.[74] To achieve non-covalent DOX loading on GO-Fe_3_O_4_, DOX is vortexed and incubated with GO-Fe_3_O_4_ conjugates overnight with no additional agitation necessary. conjugates are then separated from an unbound DOX with a strong magnet. The absorption spectra of unbound DOX remaining in the solution is used to calculate the efficiency of DOX loading (% of free DOX loaded) on GO-Fe_3_O_4_ and the loading capacity (weight percent of loaded DOX to GO-Fe_3_O_4_). Such optical approach yields high loading efficiency of 61.42% and a loading capacity of 0.2 mg of DOX per 1 mg of GO-Fe_3_O_4_ resulting in 20 wt% loading. Several works centered on DOX delivery by graphene oxide report lower or similar loading, however, show no improvement in DOX efficacy when complexed to GO.[77–79] Some can achieve substantially higher loading[80], however, do not report efficacy and in order to maintain that loading utilize GO flakes of larger sizes that may complicate cellular internalization. DOX-GO-Fe_3_O_4_ conjugates utilized in the current study in addition to drug delivery also provide the capacities for cancer detection, imaging and MRI sensing which makes the DOX-GO-Fe_3_O_4_ formulation more advantageous for theragnostic. In order to fully assess the efficacy of DOX-GO-Fe_3_O_4_ complexes we investigate both cellular internalization and cell viability in the presence of DOX-GO-Fe_3_O_4_ against DOX only control.

**Fig 7:**
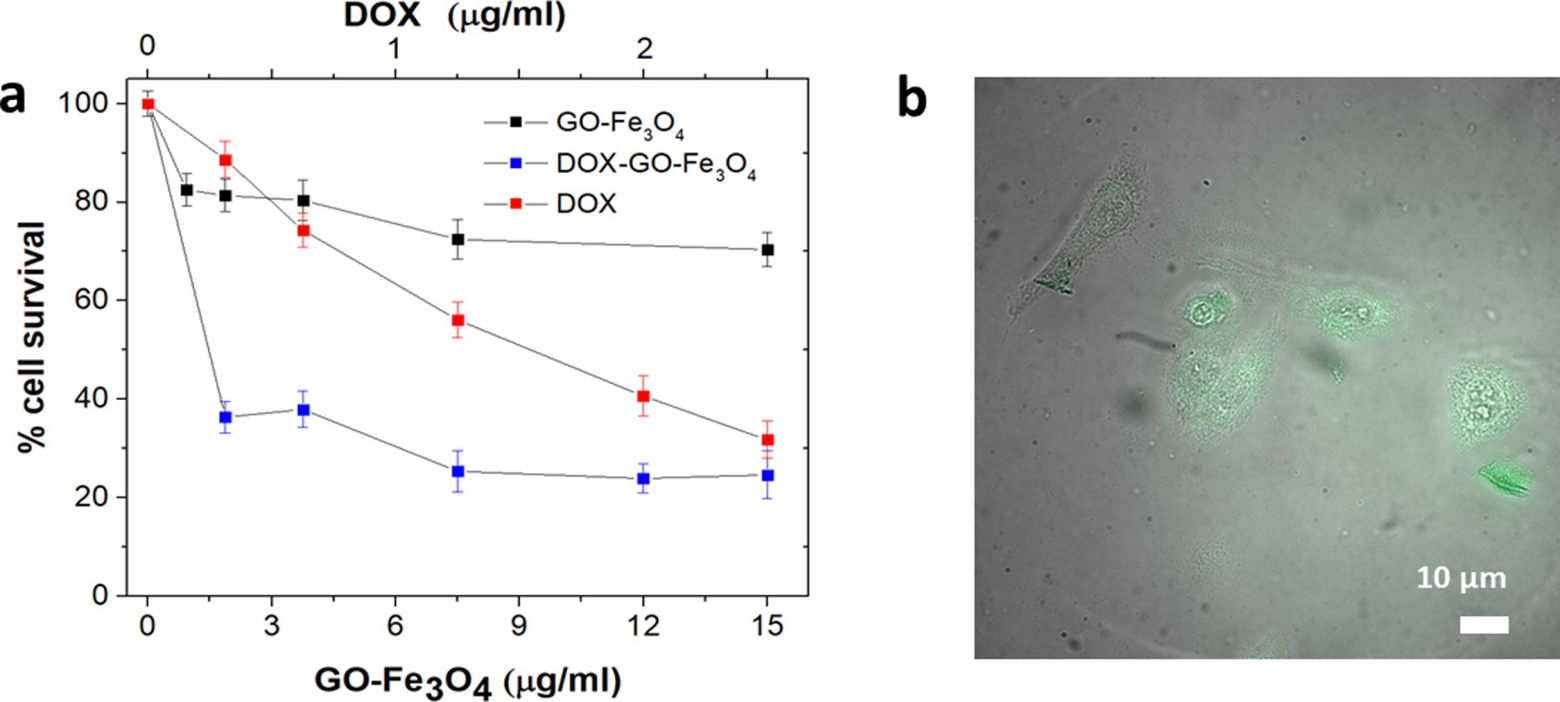
a) Cell viability of HeLa cells subject to: GO-Fe_3_O_4_ (black squares), DOX-GO-Fe_3_O_4_ (blue squares) and DOX (red squares) and b) GO-Fe_3_O_4_ internalization fluorescence imaging in HeLa cells.

Introduced to HeLa cells DOX-GO-Fe_3_O_4_ formulation shows effective internalization within the cytoplasm similarly to that of GO-Fe_3_O_4_ carriers at the 3h time point (Fig 7B). Fluorescence emission from GO-Fe_3_O_4_ platform does not exhibit significant changes due to non-covalent complexation, thus, we expect only negligible fluorescence contribution from DOX likely quenched by GO platform.

As compared to free DOX, DOX-GO-Fe_3_O_4_ conjugates provide significantly higher efficacy at lower concentrations derived from cancer cell apoptotic response (Fig 7) evaluated using an MTT assay in HeLa cells. DOX-GO-Fe_3_O_4_ offers 2.5-fold decrease in cell viability down to 37% with respect to a free drug at only ~0.3 μg/mL dose of DOX and ~2μg/mL concentration of GO-Fe_3_O_4._ The GO-Fe_3_O_4_ concentration used here is that of the whole platform. To achieve a similar response unbound DOX requires ~8-fold higher concentrations. A higher toxicity exhibited by DOX when delivered by GO-Fe3O4 can be likely explained by the improved transport and internalization with the nanomaterial delivery vehicle that is generally known to enhance the efficacy of delivered therapeutics[81–83] GO-Fe_3_O_4_ on its own exhibits only mild cytotoxicity, comparable to that of GO, which cannot account for the substantially enhanced therapeutic effect of the combined DOX-GO-Fe_3_O_4_ formulation. DOX delivery and imaging so far did not incorporate magnetic targeting that in the tissues via targeted delivery approach expected to produce higher accumulation and, therefore, further improved efficacy. This response verifies the improved GO-Fe_3_O_4_-mediated intracellular transport. An advantage of substantial loading capacity also allows to select a broad treatment range with only a small dose of nanoparticles. Although implausible in the present *in vitro* work we intend to further utilize the magnetic targeting for significantly improved delivery and efficacy[84] in the further *in vivo* studies.

## Conclusions

In this work we have successfully synthesized and tested the feasibility of multifunctional GO-Fe_3_O_4_ conjugates with capabilities of dual magnetic resonance/fluorescence imaging, magnetic manipulation for targeting, optical pH sensing and drug delivery. These novel nanoparticles have an average size of 250nm suitable for cellular internalization and show comparable to GO low cytotoxicity at imaging concentrations of 15 μg/mL. The relaxation properties of GO-Fe_3_O_4_ conjugates are comparable to existing free nanoparticle analogs, GO-Fe_3_O_4_ conjugates have potential of as negative MRI contrast agents. GO-Fe_3_O_4_ conjugates can be effectively manipulated by a magnet in suspension which allows for direct magnetic targeted accumulation in a specific therapeutic site. The GO surface contains a variety of functional groups for covalent attachment of molecular therapeutics or a substantial hydrophobic graphene platform for non-covalent functionalization with aromatic-based drugs with poor water solubility. In our work GO-Fe_3_O_4_ conjugates show efficient intracellular delivery of non-covalently attached Doxorubicin with considerable drug loading and over 2.5-fold improvement in its efficacy over free drug at low concentrations. This in turn allows using 8 times lower dose of Doxorubicin to achieve the same therapeutic effect of ~62% cancer cell death. The therapeutic delivery is tracked by the intrinsic green fluorescence of GO-Fe_3_O_4_ complex that indicates efficient internalization at 3 hours post transfection with further excretion from the cells. The pH-dependence of this emission allows using the ratios of emission intensity in green (535 nm) to red (635 nm) to differentiate between cancer (MCF-7 and HeLa) and healthy (HEK-293) extracellular environments with a substantial 4 to 5-fold difference. As a result, we propose GO-Fe_3_O_4_ as a unique multifunctional nanomaterial for magnetic-targeted drug delivery, dual *in vitro* fluorescence and *in vivo* MRI imaging and optical detection of cancerous environments.

## Supporting information

S1 Table Green/red ratios of spectral intensities for GO-Fe_3_O_4_ at different pH environments. (TIF)

S1 Fig TEM of a) GO before ultrasonic treatment: flakes sizes are in the micrometer range and b) GO after 30 tip ultrasonic treatment; average flake size is 570 nm. Right panel–histogram of GO flakes sizes after 30 min of ultrasonic treatment and c) GO- Fe3O4 size distribution with mean size of 265 nm.
(TIF)

S2 Fig UV-Vis absorption spectrum of doxorubicin (DOX). Black–spectrum of as-prepared sample with the initial concentration of DOX in water of 42 μg/mL. Red–spectrum of free DOX separated after complexation with with GO-Fe_3_O_4_.
(TIF)

S3 Fig a) 1/T1 vs iron concentration of free Fe3O4 NPs and b) 1/T2 versus iron concentration of free Fe3O4 NPs.
(TIF)

S4 Fig TEM images of GO before (a) and after (b) introduced to cell media at 37°C for 2 weeks.
(TIF)

S5 Fig (a) Size distribution of Fe3O4 NPs with an average size 5.8 ± 0.9 nm, (b) TEM image of Fe3O4 NPs and (c) HRTEM of Fe3O4 NPs.
(TIF)

S6 Fig Stability of GO-Fe_3_O_4_ in water, PBS, cell media and serum. (TIF)

S7 Fig A) DLS of GO-Fe3O4 NPs.
(TIF)

S8 Fig A) Zeta Potential GO-Fe3O4 NPs.
(TIF)
