# Supplementary figures and images for: Multifunctional graphene oxide/iron oxide nanoparticles for magnetic targeted drug delivery dual magnetic resonance/fluorescence imaging and cancer sensing
Source: PLoS One. 2019 Jun 6;14(6):e0217072. doi: 10.1371/journal.pone.0217072 (PMC6553710; doi:10.1371/journal.pone.0217072)

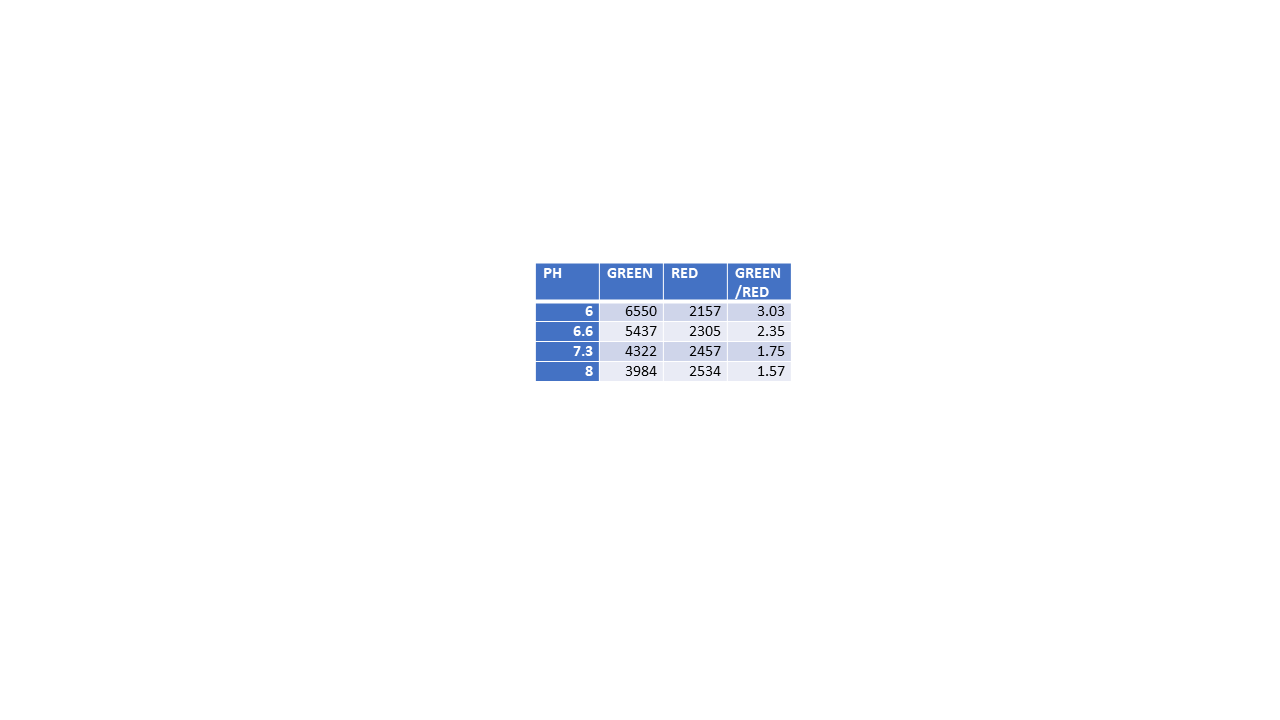

Supplement: S1 Table — (TIF) [file pone.0217072.s001.tif]

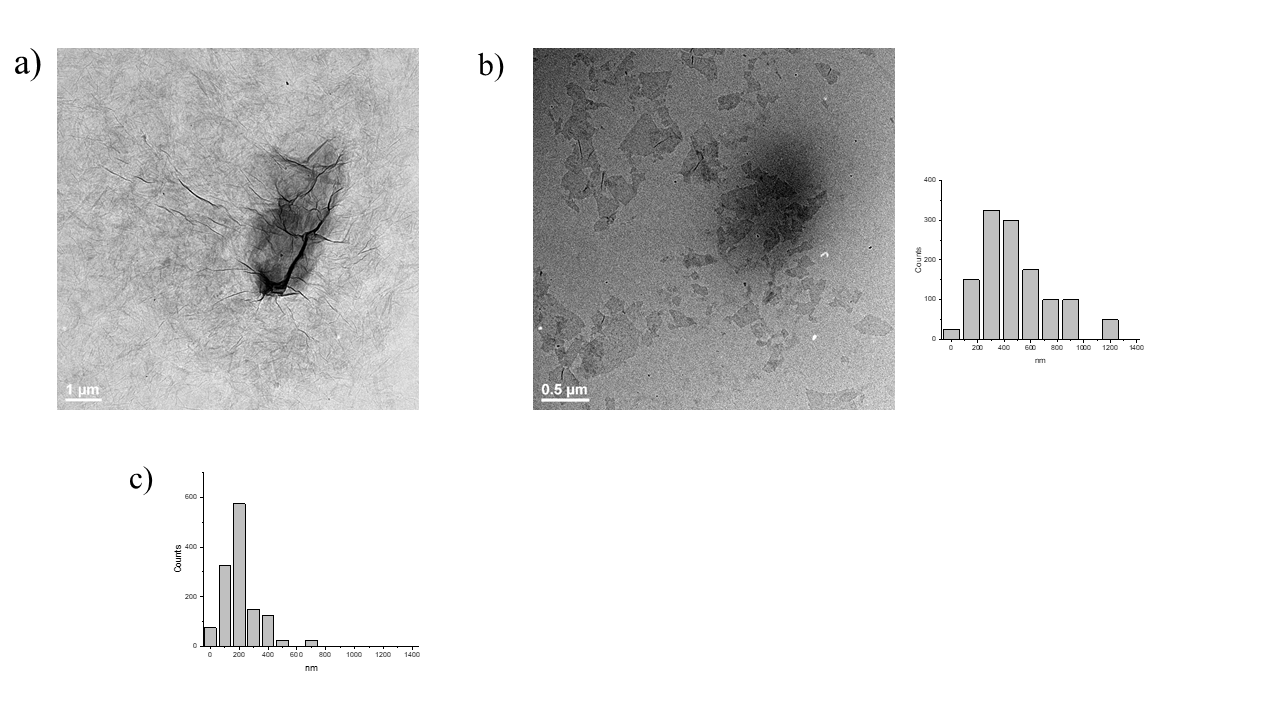

Supplement: S1 Fig — TEM of a) GO before ultrasonic treatment: flakes sizes are in the micrometer range and b) GO after 30 tip ultrasonic treatment; average flake size is 570 nm. Right panel–histogram of GO flakes sizes after 30 min of ultrasonic treatment and c) GO- Fe3O4 size distribution with mean size of 265 nm. (TIF) [file pone.0217072.s002.tif]

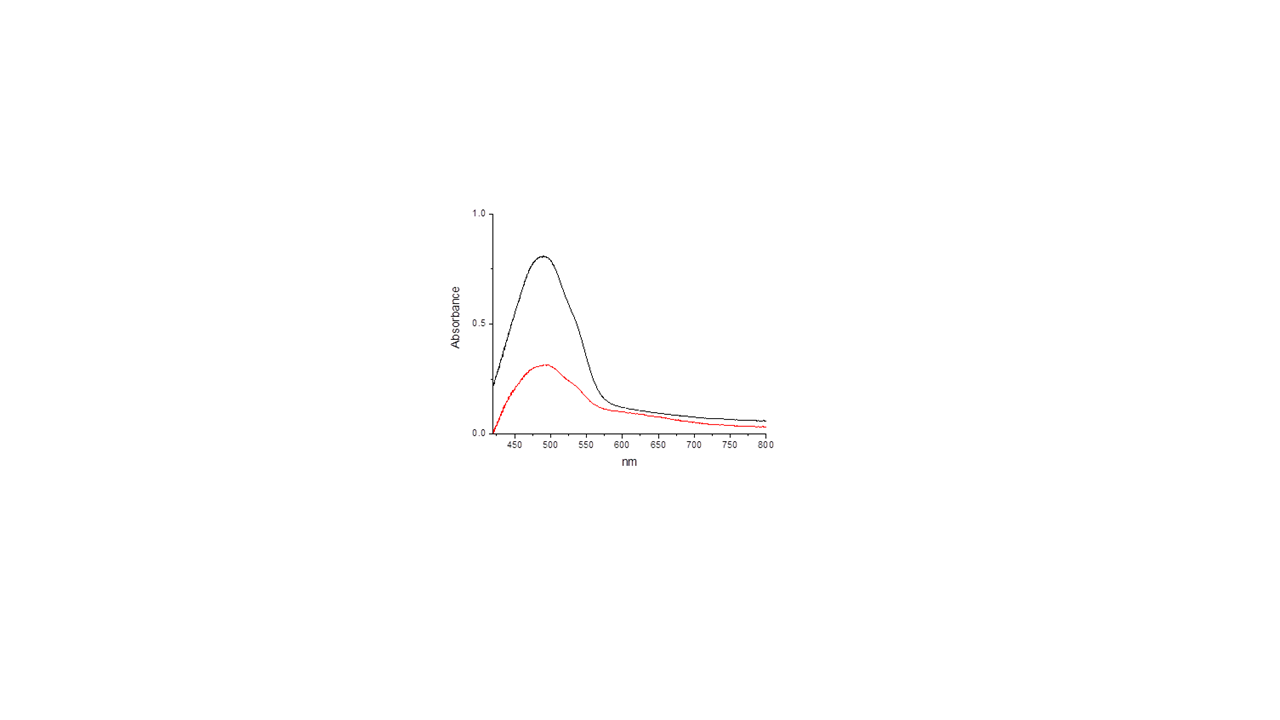

Supplement: S2 Fig — Black–spectrum of as-prepared sample with the initial concentration of DOX in water of 42 μg/mL. Red–spectrum of free DOX separated after complexation with with GO-Fe3O4. (TIF) [file pone.0217072.s003.tif]

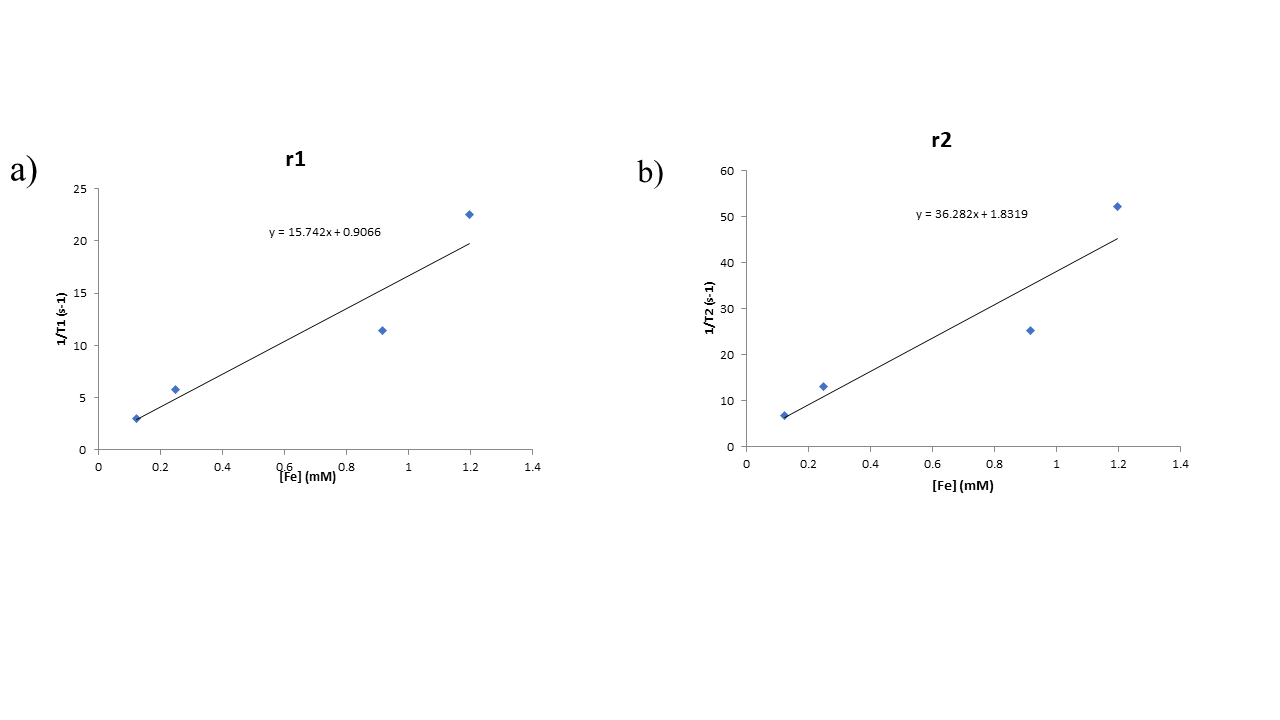

Supplement: S3 Fig — a) 1/T1 vs iron concentration of free Fe3O4 NPs and b) 1/T2 versus iron concentration of free Fe3O4 NPs. (TIF) [file pone.0217072.s004.tif]

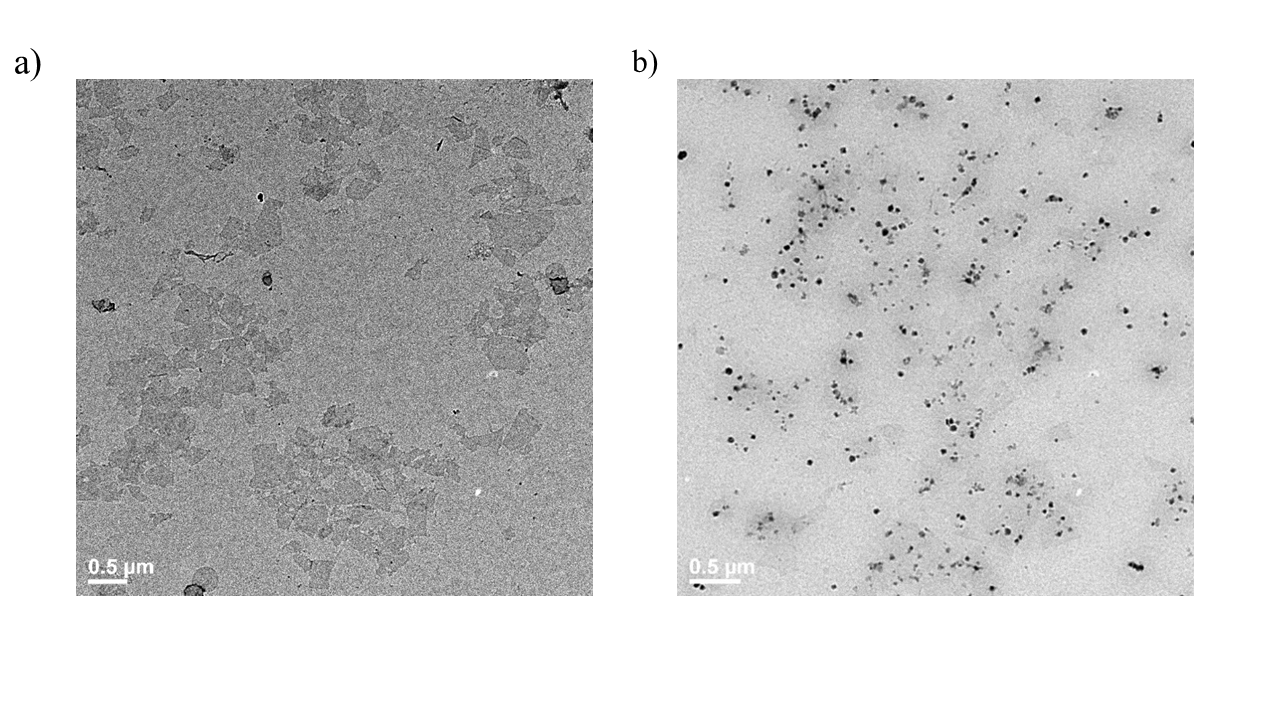

Supplement: S4 Fig — TEM images of GO before (a) and after (b) introduced to cell media at 37°C for 2 weeks. (TIF) [file pone.0217072.s005.tif]

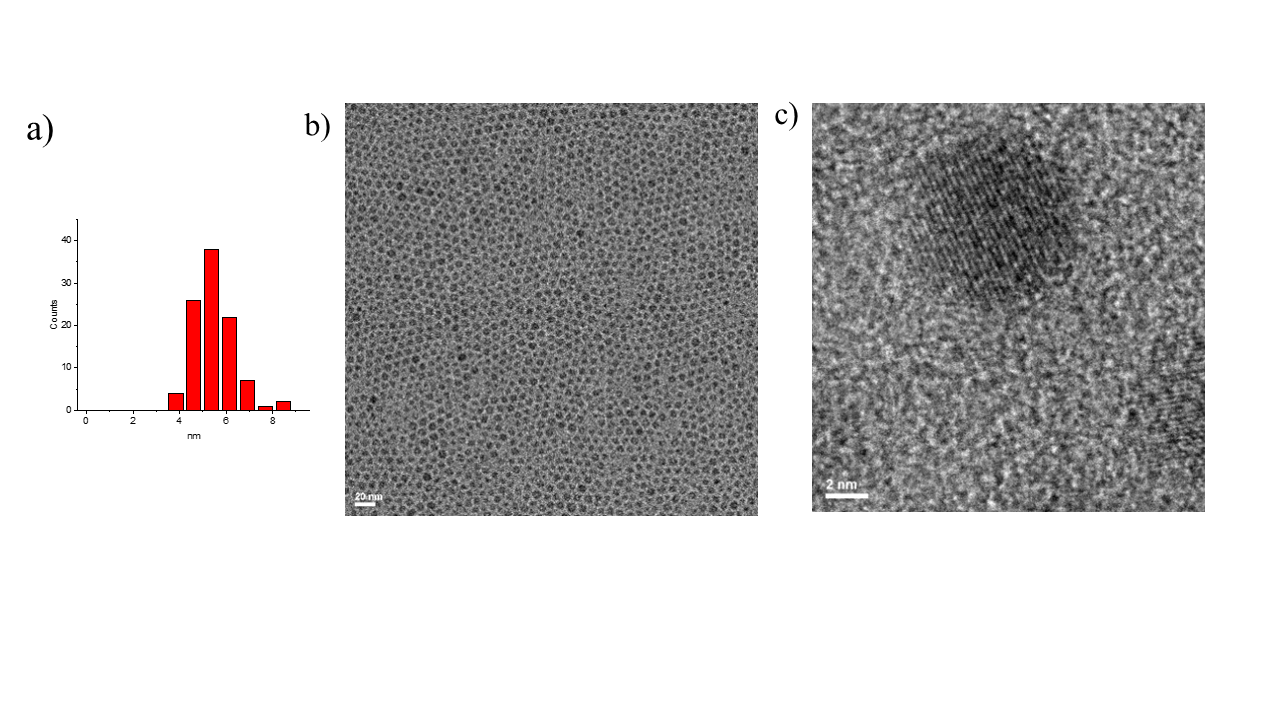

Supplement: S5 Fig — (a) Size distribution of Fe3O4 NPs with an average size 5.8 ± 0.9 nm, (b) TEM image of Fe3O4 NPs and (c) HRTEM of Fe3O4 NPs. (TIF) [file pone.0217072.s006.tif]

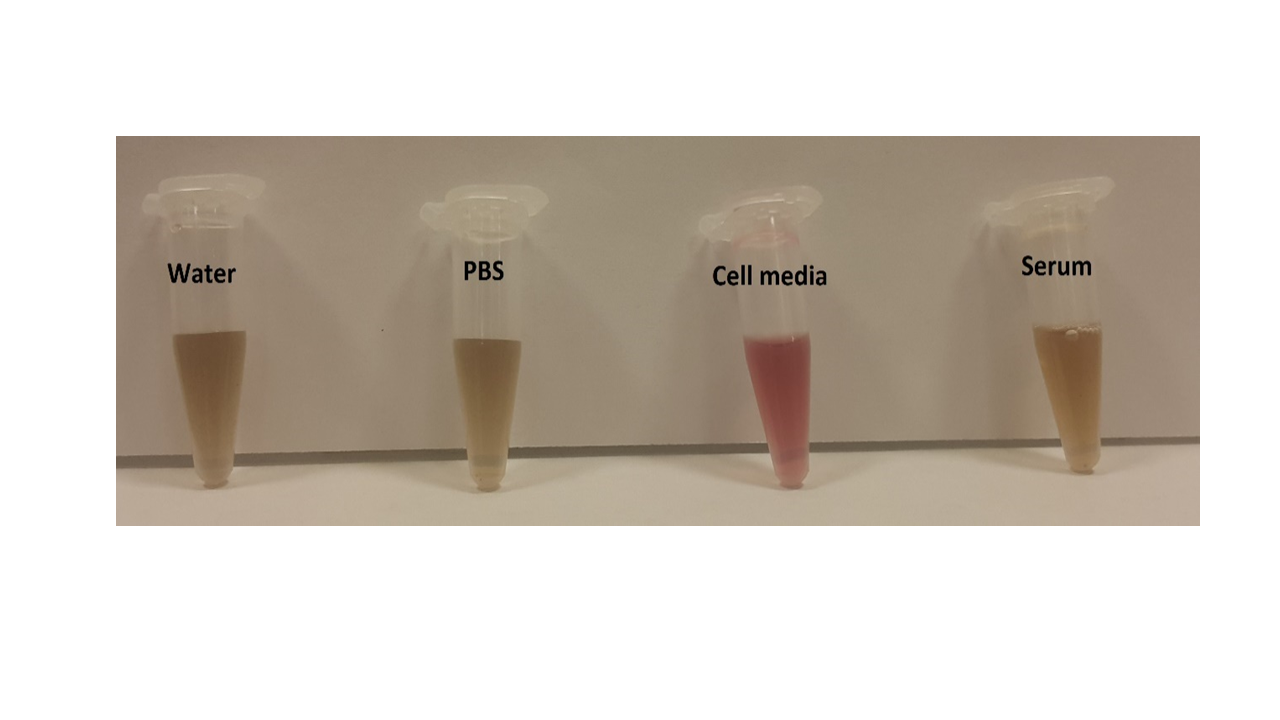

Supplement: S6 Fig — (TIF) [file pone.0217072.s007.tif]

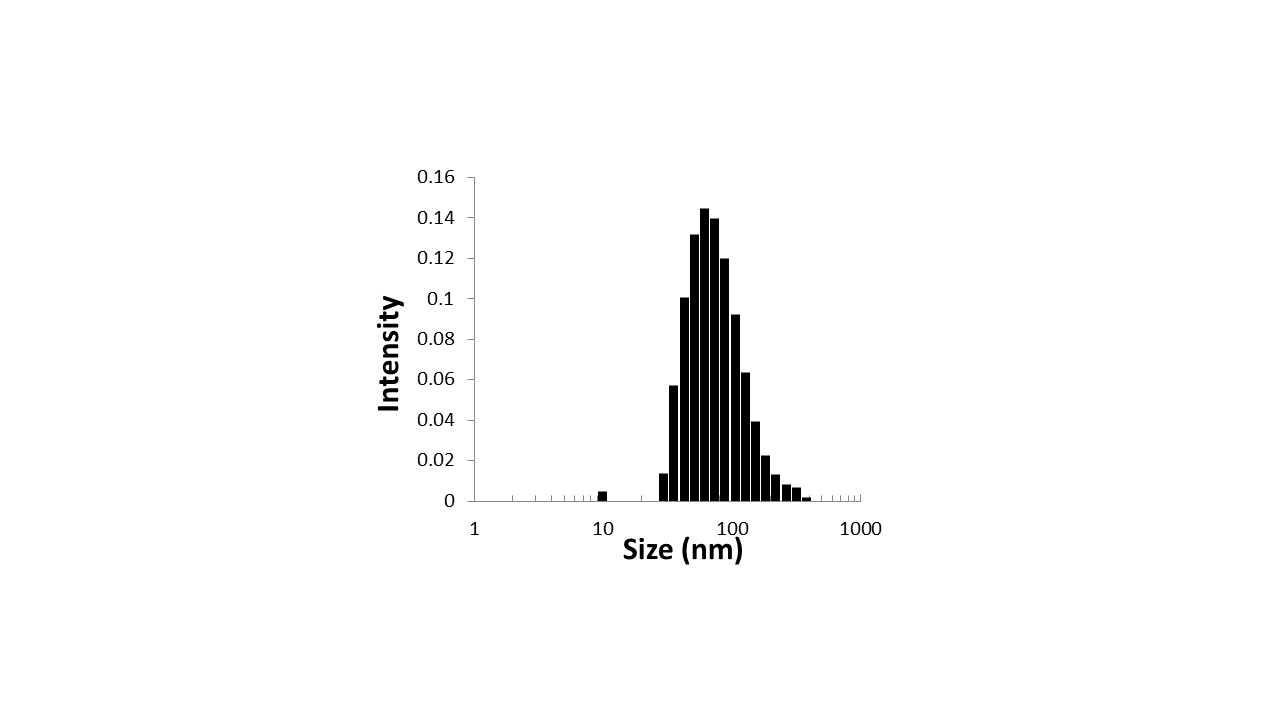

Supplement: S7 Fig — A) DLS of GO-Fe3O4 NPs. (TIF) [file pone.0217072.s008.tif]

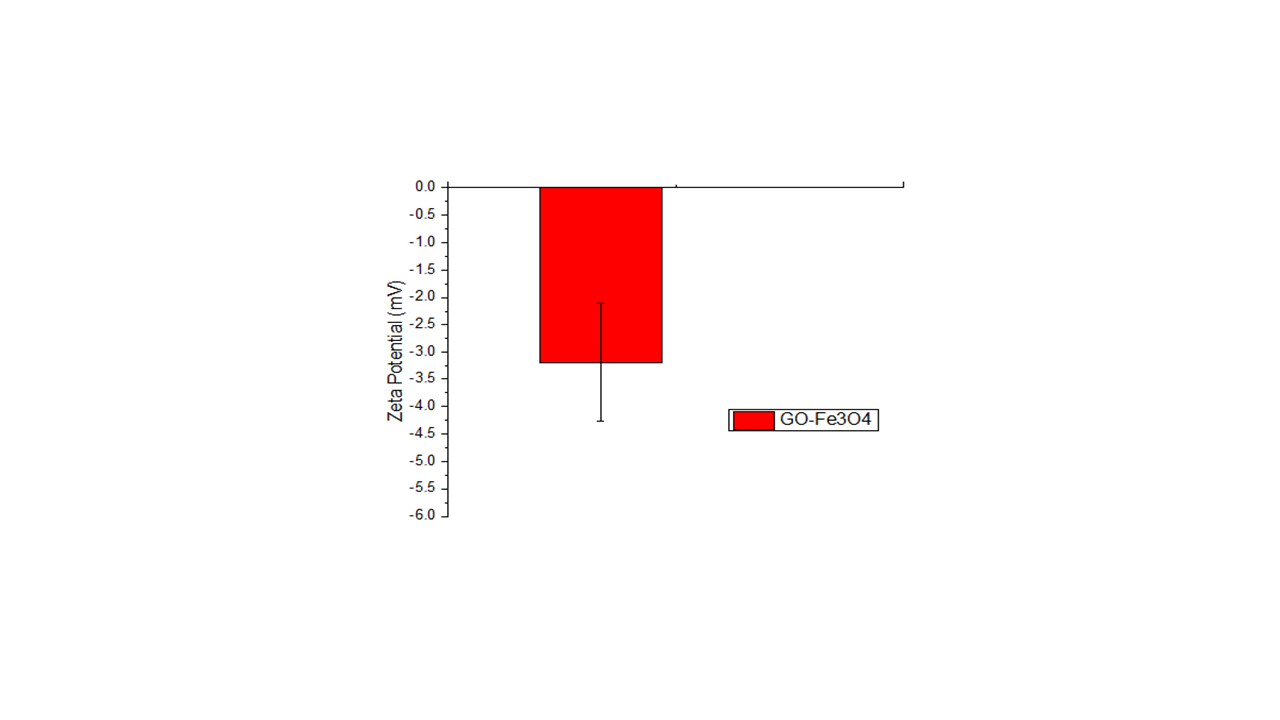

Supplement: S8 Fig — A) Zeta Potential GO-Fe3O4 NPs. (TIF) [file pone.0217072.s009.tif]
